# Supplementary material for: Conditional chemoconnectomics (cCCTomics) as a strategy for efficient and conditional targeting of chemical transmission
Source: eLife. 2024 Apr 30;12:RP91927. doi: 10.7554/eLife.91927 (PMC11060718; doi:10.7554/eLife.91927)
Supplement: Figure 3—source data 3. [file elife-91927-fig3-data3.zip › Figure 3 source data3 part 2.zip!/Figure 3-Source data 3-part2/1002-9-CRISPResso_Report_e53dd8m5.zip!/CRISPResso_on_1002_12/1c.Alignment_barplot.pdf]

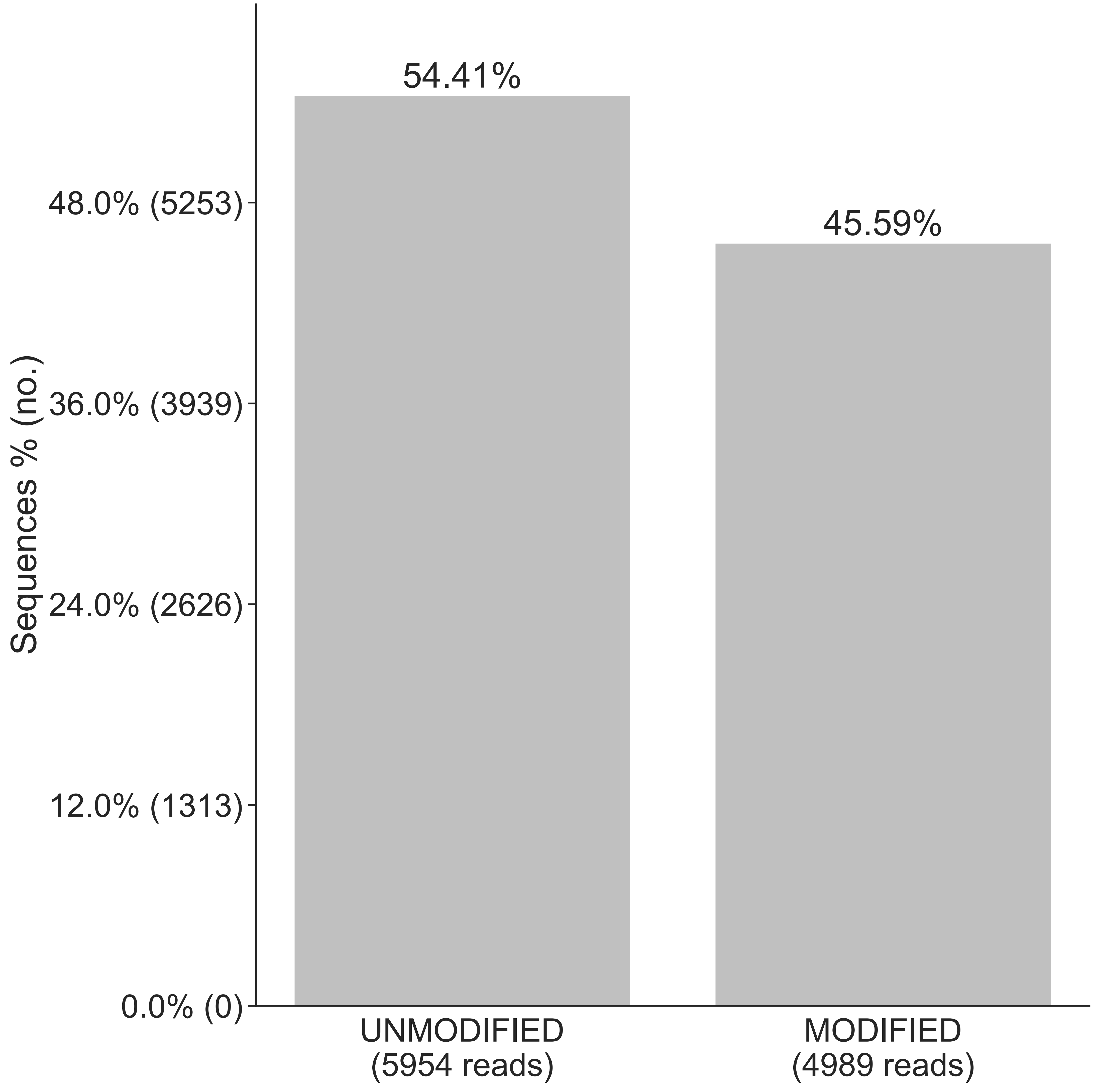

54.41%
45.59%
Sequences % (no.)
48.0% (5253)
36.0% (3939)
24.0% (2626)
12.0% (1313)
0.0% (0)
UNMODIFIED
(5954 reads)
MODIFIED
(4989 reads)
